# Residents’ Perceptions of a Community-Led Intervention on Health, Well-Being, and Community Inclusion Through Photovoice

**DOI:** 10.1177/10901981211009738

**Published:** 2021-05-21

**Authors:** Clare Jackson, Sara Ronzi

**Affiliations:** 1 Blackpool Council and Lancashire Violence Reduction Network, Blackpool, UK; 2 London School of Hygiene and Tropical Medicine, London, UK; 3 University of Liverpool, Liverpool, UK

**Keywords:** community-based participatory research, evaluation, health, photovoice, social inclusion, UK

## Abstract

Community-centered approaches can be effective ways to engage communities and improve their health and well-being. The Grange is a community-led, multifaceted, and dynamic intervention incorporating a community hub and garden, that took place in a small area of the North-West of England, characterized by high levels of deprivation and poor health. Activities have been defined, developed, and supported by residents to meet locally defined needs. This study used photovoice methods to explore residents’ perceptions and experiences of this community-led intervention and any perceived impact on health, well-being, and community inclusion. Through photographs, semistructured interviews, a focus group discussion, and an exhibition, this study engaged intensively and creatively with a group of six residents. They identified positive and negative aspects related to The Grange and suggested recommendations that were directly communicated to policy makers during the photo-exhibition event. Participants reflected on various activities such as the community garden and the community shop. They also reflected on contextual factors and suggested that the culture of inclusivity and friendships associated with The Grange were more important to them than specific activities. This study demonstrated the value of using photovoice to (a) explore residents’ perceptions of community led interventions; and (b) meaningfully engage residents living in areas with high levels of deprivation. Public health practitioners should consider the use of photovoice (a) in the evaluation of health interventions that take place in a complex and changing context, and (b) as a powerful tool to engage with members of the community, especially traditionally disadvantaged groups, to ensure that engagement about health, well-being, and social inclusion is meaningful.

Despite recognition of great health inequalities for socioeconomically disadvantaged groups in the United Kingdom (Marmot et al., 2020), health is still persistently poorer in the North, with the gap widening over the past 40 years (Hacking et al., 2011). People living in the North of England are especially disadvantaged by a range of factors including power, poverty, damaging environments and conditions, and resources needed for health (Whitehead, 2014). The recurrent cuts and reduced capacity within local government (The King’s Fund, 2018) and the complex systems within which health inequalities are ingrained (Rutter et al., 2017) make it increasingly important to harness community assets—both physical and skills based—through community-centered practice (Davis et al., 2019).

Community-centered approaches, a family of nonclinical interventions that draw on community assets (Public Health England & NHS England, 2015), can be effective ways to improve health and well-being through the mobilization and empowerment of individuals, to address both behavioral and structural factors, which impact on health (Public Health England, 2018; NHS England, 2016). Among their potential benefits is the narrowing of health inequalities through engaging with and hearing the voices of the most disadvantaged, and increasing access to a range of health promoting services (Marmot et al., 2020). Furthermore, increasing the conditions for people to take control over their lives (Morgan & Ziglio, 2007) has been advocated for its potentially transformative impact on population health (Bagnall et al., 2018; O’Mara-Eves et al., 2013; Orton et al., 2017; Wallerstein, 2006; Whitehead et al., 2016). Community-led interventions, such as The Grange, sit within the wider “family” of community-centered approaches (Public Health England & NHS England, 2015).

## Background

### Intervention Description

“The Grange” is a community-led garden and hub situated in Blackpool, North-West England, an area characterized by long-standing health inequalities, with both general health and life expectancy significantly worse than the United Kingdom (Public Health England, 2020). Developed in 2017, residents, third sector organizations, and Blackpool Council commenced a program to develop a previously unused “City Learning Centre.” The program moved local shops from buildings that were no longer “fit for purpose,” and utilized the hub to create “opportunities for health and community cohesion” among residents and to support wider developments on the estate (Blackpool Council, 2017b, p.2). The Grange can be defined as a community-led intervention because its activities have been defined, developed, and supported by residents through a “bottom up” governance model. Through this model, The Grange places local people at the heart of decision making and ensures that The Grange is able to respond to locally defined needs. The activities are interwoven within a building and community garden, aimed at all ages, from children to the elderly, with different needs and issues (e.g., mental health problems). The community garden enables residents to grow fruit and vegetables, which are sold within the community shop. Other activities include a library, volunteering programs, time banking for shopping credits in the community shop, community café with weekly free family meals, and adult learning programs (Groundwork, 2020; see Supplemental Appendix A).

The Grange can be considered an example of a complex public health intervention as it is formed by interacting components requiring input from multiple stakeholders which responds flexibly to the changing users’ needs and context (Craig et al., 2018; Hawe et al., 2004; Petticrew, 2011). Evaluation of these interventions, especially natural experiments, can be challenging as they are context-specific, adaptable, and not under the control of the researcher (Craig et al., 2012). Public health research has evaluated natural experiments for many decades, given that many interventions do not lend themselves well to more traditional study types such as randomized-controlled trials (Craig et al., 2017). Qualitative methods and community-centered approaches—intended to facilitate participation and action through participants’ collaborative efforts (Davis et al., 2019), can, however, help understand the factors that determine exposure or affect intervention design and delivery (Craig et al., 2018; Moore et al., 2019).

Since the creation of The Grange, informal conversations with the center staff and Blackpool Council officers (C. Jackson, personal communication, December 2019) suggested (a) a positive impact on community engagement, social isolation, and a reduction in antisocial behavior and arson incidents on the local housing estate; and (b) an active engagement of local residents in the hub, where past interventions delivered in the area failed to do so. For instance, residents had failed to engage with the café and adult learning offer within the previous City Learning Centre. Reasons included viewing the offer as being “too corporate and somewhat intimidating” and “lack of trust in statutory bodies such as the Council or the Police, where activities were felt to be imposed in residents, or not maintained for the longer term” (C. Jackson, personal communication, December 2019).

No formal studies of The Grange have been undertaken. There was an identified desire to explore the ways through which The Grange managed to engage meaningfully the residents, and to explore what the perceived impact has been on health, well-being, or community inclusion. The complex nature of The Grange, both in terms of the context and the coproduction of the offer, underpinned by a “bottom-up” governance model, makes it an important public health intervention to be investigated. It offers an opportunity to further enhance the field of community health promotion through understanding residents’ perceptions of community-led initiatives.

### Study Aim

This study aimed to explore residents’ perceptions and experiences of this community-led intervention and any perceived impact on health, well-being, and community inclusion. Furthermore, by adopting a community-based participatory research (CBPR) approach, this study aimed to actively engage participants in the research, to identify community recommendations, and to engage participants in communicating their views about The Grange directly to local “influential advocates.”

## Method

### Study Context

This study took place in the North-West of England. Grange Park has a population of approximately 6,000 and experiences high levels of socioeconomic deprivation: 45.5% children aged 0 to 15 years live in households with lower socioeconomic status compared with 19.9% nationally; 7.6% of residents claimed Job Seekers Allowance, significantly higher than the England average 3.8%; the rate of long-term unemployment is 2.4% compared with the England average 1% (Blackpool Council, 2017a). Moreover, self-reported health and adult education are all significantly generally worse than the England average. However, Grange Park Estate has also many assets including large green spaces and generally low transience compared with the rest of Blackpool, which experiences high levels of population movement (Blackpool Council, 2017a).

### Photovoice Methods

This study adopted a photovoice methodology within a CBPR approach (Israel et al., 2010; Minkler, 2004) to explore residents’ perceptions and experiences of this community-led intervention and any perceived impact on health, well-being, and community inclusion. CBPR is a research approach, which comprises participation, action, and collaborative inquiry, often employed to solve public health problems and benefit community health (Minkler, 2005).

Originally created by Wang and Burris (1997), photovoice uses photographs taken by participants that represent issues that are important to them. It is grounded in Freire’s (1970, 1974) concept of critical consciousness whereby photographs are an important visual stimulus for (a) individual reflection about their community and (b) advocating for social or political change. Photovoice seeks to enable participants to become conscious of their perceptions and community issues and to further frame and define these perceptions through focus group discussions (FGDs; Carlson et al., 2006).

We considered photovoice as a suitable approach for several reasons. First, it can be considered an inclusive research approach as visual images can aid those with lower literacy skills to participate in research (Hergenrather et al., 2009). It also allows participants greater opportunity to shape the focus of the research—as the data collected depend on the photos they produce and the meanings they have about those photographs (Catalani & Minkler, 2010). Second, photography can assist to collect contextualized information that might otherwise be inaccessible to researchers using solely interviews or FGDs (Liebenberg, 2018). Third, photovoice aims to drive positive change and implementing this method in areas of high disadvantage such as Blackpool has the potential to reduce health inequalities through enabling participants to voice their perceptions to decision makers and influence future service delivery (Catalani & Minkler, 2010). Moreover, CBPR approaches not only extract data to the benefit of the researcher or wider organization(s) but can add local value for participants and the wider community such as skills, confidence, or empowerment (Budig et al., 2018), which are recognized to reduce inequalities (Whitehead, 2014).

### Participants and Recruitment

We used a purposive sampling strategy to recruit participants with a range of demographic backgrounds including gender, education level, employment, age, and family circumstances so as to gather a range of views and experiences (Table 1). We aimed to recruit adults who (a) were aged older than 18 years, (b) lived in the local housing estate, (c) spoke English, and (d) were both users and nonusers of The Grange. While a gatekeeper working at The Grange facilitated recruitment, the first author frequently visited the hub during the recruitment process to build rapport with participants and observe group dynamics (Abma et al., 2019).

**Table 1.** Participant Demographics.

ID	Gender	Age (years)	Ethnicity	Occupation	Highest educational Qualification	Marital status	Household makeup	How often attends the Grange?	Undertakes volunteering at The Grange
1	Female	75	White British	Retired	GCSEs or equivalent	Single	Lives alone	Several times per week	No
2	Male	52	White British	Unemployed	No qualifications	Single	Lives alone	Several times per week	Yes (Garden)
3	Female	59	White British	Unemployed	GCSEs or equivalent	Divorced	Lives alone	Several times per week	Yes (Garden)
4	Male	51	White British	Volunteer	No qualifications	Single	Lives alone	Several times per week	Yes (Garden)
5	Female	44	White British	Housewife	No qualifications	Single	Lives with 5 children	Every day	Yes (Community Shop)
6	Female	26	White British	Volunteer	GCSEs or equivalent	Single	Lives alone	Several times per week	Yes (Community Shop)

*Note.* GCSE = General Certificate of Secondary Education.

### Procedures

We slightly modified the methodology by Wang and Burris (1997), which uses FGDs, by adding an individual semistructured interview (SSI; Table 2), as participants were likely to know each other due to living in the same community, and therefore might have found it difficult to express honest views of The Grange solely through FGDs. The SSI explored (a) the meaning and importance of the photos and (b) the participant’s views of The Grange (see Supplemental Appendix B). The topic guide was refined iteratively during data collection, to account for interesting phenomena discussed in the SSIs (Agee, 2009). The FGD session, instead, aimed to stimulate discussion about the participants’ photographs (Wang & Burris, 1997).

**Table 2.** Photovoice Procedures of This Study.

Stage of the research process	Activities
1: Initial training meeting	• All participants signed a written informed consent form prior to participation. • Participants attended a meeting at The Grange, which lasted around 1 hour. • The research team introduced participants to the study, what we wanted them to capture with the photographs, and how the photographs would be used in the study (e.g., photo-exhibition). • Participants received training in using digital cameras. They could choose to use their own camera or phone, or to borrow a camera. Two participants decided to use the camera on their mobile phone, four participants chose to borrow a digital camera. • Ethics training: participants were explained when to gain consent from people who might feature in their photographs. The training ensured that participants understood the “rules” on when consent was needed (e.g., individual or group is “featured”), and when not (where people can be regarded as a crowd). We advised participants not to take photographs of people younger than 18 years unless they were family members or friends (Wang & Redwood-Jones, 2001).
2: Photo-taking task	• Over 10 days, participants took photographs of “any object/person/aspect that represented their views of The Grange, its impact on health, well-being, and their experiences of feeling part of the Grange Park community.” • Participants were encouraged to consider aspects of The Grange that worked well and things that could be improved. • Participants took an average of 12 photographs each.
3: Individual SSIs	• Participants took part in a SSI in a private meeting room at The Grange, where they discussed approximately six of their photographs that they thought best represented the study topic. Choosing six photographs allowed an in-depth discussion of the photos • Interviews lasted approximately from 1 hour (from 35 minutes the shortest to 1 hour and a half the longest; topic guide in Supplemental Appendix B). • Interviews were audio recorded with participants’ permission. • Based on best practice in photovoice, we incorporated the SHOWeD questions in the SSI as prompts to help participants discussing the photographs (What do you *See* here? What’s really *Happening* here? How does this relate to *O*ur lives? *W*hy does this problem or this strength exist? What can we *D*o about this? Wang et al., 1998). • The interview also explored the participant’s experience with photovoice and any photographs that the participant might have wanted to take but you did not take (Hodgetts et al., 2007).
4: Focus group discussion and participatory data analysis	• Participants attended a focus group discussion, where they discussed their photographs together. This took place in a meeting room at The Grange. • This session lasted an hour and 15 minutes and was audio recorded with participants’ permission. • Later, participants grouped their photographs into common clusters and developed themes that emerged from the photographs and discussion (further discussed in Section 2.6). • Participants generated six themes including focal points and gathering places; gardening as a therapy; “forget your troubles and feel safe”; not being judged; environmental sustainability; and involving the community in meaningful activities (Supplemental Appendix E). • They then agreed on a list of key findings and recommendations that they wanted to feedback to senior decision makers across Blackpool during the exhibition event (Phase 5). • Participants wrote the title of each of the photo they wanted to see displayed at the exhibition.
5: Photo-exhibition event and engagement with policy makers and influential advocates	• Participants disseminated the findings in a photo-exhibition event at The Grange hub with policy makers and influential advocates in Blackpool. • Five participants (out of six) and five influential advocates attended the event, including representatives from Blackpool Council (commissioners, Public Health Consultant and Research Department), and The Grange management. • The session involved an informal discussion between participants and influential advocates about their photographs, key priorities, and recommendations for The Grange and Blackpool Council. • A total of 36 photos were displayed (each participant had 6 photos displayed). • Captions accompanying each photo were prepared based on the interviews’ transcripts and checked by participants for any modification and approval (Evans-Agnew & Rosemberg, 2016). • The event was assessed through a brief evaluation to record what participants enjoyed about the experience, what they had learned, and so on. • The Grange staff and participants created a permanent photo-exhibition at the hub (from August 2019) to disseminate more widely the findings.

*Note*. SSI = semistructured interview. Adapted from Wang and Burris (1997), Wang et al. (1998), Ronzi et al. (2016), Nykiforuk et al. (2011).

Wang and Burris (1997) set out three overarching goals of photovoice that are facilitated through different stages of the research process (Table 2). First, to enable people to reflect on their community (Stage 2 photo-taking task; Stage 3 SSIs; Stage 4 FGD). Second, to promote critical dialogue about important issues through FGDs (Stage 4 FGD). Third, to reach policy makers and advocate for change (Stage 4 FGD; and Stage 5 photo-exhibition).

### Ethical Considerations

The study obtained ethical approval by the Ethics Committee of The University of Liverpool. Photovoice presents additional ethical considerations related to the use of photographs. Following ethics guidance (Evans-Agnew & Rosemberg, 2016; Wang & Redwood-Jones, 2001) participants received two additional written consent forms (Table 2): (a) the acknowledgment and release form—for people appearing in any photograph, and (b) photograph release form asking permission to use participants’ photographs in dissemination of results. These forms ensure that participants take consent before taking photos of individuals and acknowledge that the participants are the owners of the photographs. At the end of the study, participants received a £10 “Love 2 Shop” voucher as a thank you for their commitment to the research.

### Data Analysis

We anonymized transcripts by replacing participant names with an ID number (e.g., P2). We used inductive thematic analysis to analyze the data (Braun & Clarke, 2006). After multiple readings of transcripts, the first author created a list of “data driven” codes applied across all transcripts, ensuring that codes identified in later scripts were applied to those previously coded. The inductive nature of code generation allowed for themes to be interpreted from the data. Codes were later grouped in natural clusters and these categories labelled accordingly to create the analytic framework (see Supplemental Appendix C). Last, further analysis of codes and categories led to the interpretation of themes that were supplemented by those generated by participants during the FGD (see Supplemental Appendix D; Drew & Guillemin, 2014). Twenty percent of the transcripts were line-by-line coded independently by the last author to check for accuracy, any conflict that arose was resolved through consensus.

Wang and Burris (1997) emphasize that the meaning of the photographs can only be analyzed through the ways in which participants interpret them. During data analysis, photographs were considered within the context of the corresponding transcripts and not analyzed separately. Analysis involved triangulation of themes identified from coded transcripts, participants’ interpretations of photographs, and participant-generated themes during the FGD (Ronzi et al., 2019). Supplemental Appendix D provides an example of how the different data elements are linked.

## Findings

Six residents participated in this study (demographic details in Table 1) and discussed a total of 36 photos. While the sample could be viewed as somewhat homogeneous (all White British, single/divorced and mainly live alone), it is reasonably representative of the ethnic and demographic make-up of residents living in the local area (Blackpool Council, 2017b).

From the data, we identified an overarching theme that underpins all the other themes. This includes a sense of equality, coproduction and power balance among volunteers, staff, and users in decision making and running of the project, including not feeling judged and being given responsibility. The combination of these aspects created a sense of inclusivity, which made participants feel valued and led to increased confidence. The following sections present findings on the four themes that were identified.

### Giving and Receiving Help and Support

The Grange represented a place that participants could turn to for help if needed, which proved very valuable, particularly during times of adversity.

Just before Christmas, my benefits stopped. I was absolutely devastated, I couldn’t cope, I didn’t know where to go, who to turn to. [The Grange Staff] helped me a lot and put me in touch with a lot of places. They kept me going [ . . . ] if it wasn’t for them, I wouldn’t have come back, even now. (P3, Female, 59 years)

**Figure 1.**
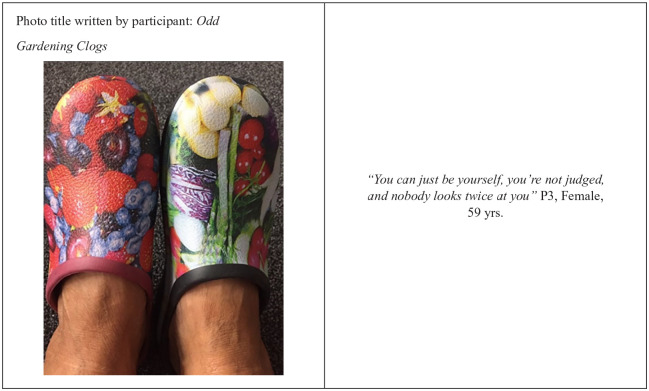
Photovoice picture by P3.

According to participants, providing support, together with a sense of not feeling judged, were all elements of the ethos of The Grange that contributed to their sense of inclusion (Figure 1 ).

The Grange has a community shop within the hub—stocked with donations and products produced in the community garden—where anyone can purchase items with money or volunteer credits, earned through volunteering at The Grange. Given the challenges for some participants in finding employment (mainly due to personal circumstances and mental health issues), volunteer credits represented a source of financial support that some participants used in the shop to buy food.

I’m on sick leave for depression [but] the benefits . . . [ . . . ] you don’t get a lot [ . . . ]. The good thing about The Grange is that for volunteering [ . . . ] you earn points [for] food [ . . . ] and that helps me immensely. (P3, Female, 59 years)

However, volunteer credits were not merely perceived as financial aid but as contributing to their feeling of control, choice, and deserving. This was in contrast with other forms of support that they felt stigmatizing (e.g., food banks). Regular use of the community shop and familiarity of the staff seemed to make its use more acceptable to participants.

P4: “[ . . . ] the shop does really help during the week, when you haven’t got enough money . . . [you can] use your points to buy food”

Facilitator: “What would you do if you didn’t have the provision shop?”

P3: “I’d be lost”

P4: “I wouldn’t use the food bank . . . I don’t know, there’s something about food banks”

P2: “ . . . you feel guilty”

P4: “Yeah, I would feel really guilty”

P5: “[ . . . ] sometimes it’s a bit intimidating as well, isn’t it?”

P3: “Yeah”

P5: “But somewhere like the shop, they make you feel welcome in there . . .”

P4: “That’s because we know them so well . . .”

(Focus Group discussion)

Interestingly, when probed further, none of the participants had ever used a foodbank. Perceptions of food banks might have been based on word of mouth, others’ experiences, or views published by media.

Others, instead, used their volunteering credits to help others in need, contributing to their sense of community inclusion.

I don’t use the provision shop; I just do it [volunteering] because I want to do it [ . . . ] but I save my points up [ . . . ]. I’m going to be doing hampers, and it will be going to people who need it. If I needed it, I’d gladly use the shop. (P5, Female, 44 years)

### The Grange: A Safe and Supportive Space to Interact With Others and Feel Part of a Community

**Figure 2.**
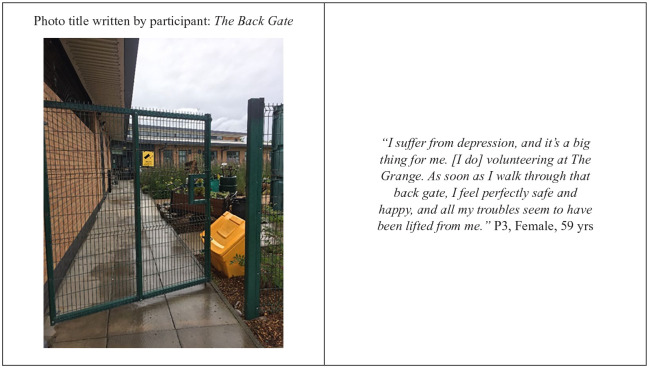
Photovoice picture by P3.

Through providing activities and volunteering opportunities, The Grange provided somewhere for residents to “get out of the house” and “escape” from their everyday routine. Doing so seemed to reduce participants’ feeling of isolation and positively contribute to their mental well-being (Figure 2) .

I remember when somebody brought me down just to show [The Grange] to me . . . I noticed from not doing very much, to coming down to the garden. It made a difference. (P2, Male, 52 years)

[by] coming in here, you just seem to forget everything. [all the troubles]. (P5, Female, 44 years)

Four participants described the importance of identity both collectively as members of The Grange, and as individuals with a defined role (e.g., garden volunteer). The objectives and identity of The Grange—which they helped shape—gave participants something to belong to, enhancing their feelings of inclusion.

P1: . . . I know what I belong to now. It’s not just a building.

P5: It just changes it for me from just [being] a building . . . to make you want to come back in here more [ . . . ]. You’re part of the team, you’re not just a volunteer, you’re part of [The Grange]. (Focus Group Discussion)

Moreover, The Grange created a safe and supporting space where participants felt comfortable to interact with others and part of a community.

For the last 20 years, I’ve never had any friends whatsoever, never went out anywhere . . . [ . . . ], I’ve always wanted to be part of a community, and now, I feel like I’m part of one. (P3, Female, 59 years)

I’ve actually stayed in my room for over 5 years. I did go out at times, but I kept getting bullied. But here, you [ . . . ] feel included in the group. [The Grange] makes you feel like you’re part of a team. (P6, Female, 26 years)

### The Hub and Garden and Their Perceived Impact on Health, Well-Being, and Community Inclusion

**Figure 3.**
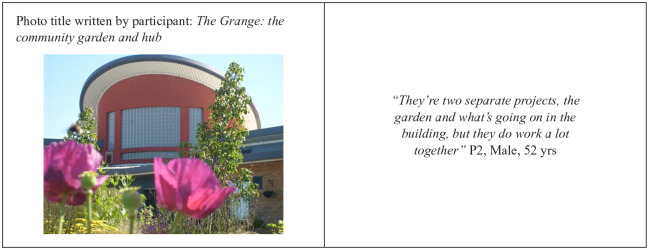
Photovoice picture by P2.

Because of their personal interests and involvement in certain activities, some participants described The Grange as having two separate identities: the community garden and the hub (Figure 3). For those interested in the garden, community gardening not only offered an opportunity to interact with others but also affected their physical and emotional health.

[when] I start weeding on the ground, just by myself, I feel great inside. [ . . . ] I don’t know why plants and gardening really help you, but it has really helped me. I really enjoy it. (P4, Male, 51 years)

Further, the ability of the garden to produce fresh fruit and vegetables for sale was a wider benefit that facilitated access to affordable healthy food.

It is very difficult [to get hold of healthy food around here]. If you go into the local shop, they have a very limited supply of fresh fruit and vegetables . . . So, you’ve got to travel. If you haven’t got transport, going on the bus to the supermarkets can be expensive. I think it’s important that there is local, food, fresh products here at The Grange. (P1, Female, 75 years)

Others, instead, saw The Grange as an intervention made of composite parts.

According to some participants, the dynamic relationship between the activities in the inside (hub) and outside (community garden) maximized the benefits felt to health, well-being, and confidence.

The inside encourages you to go outside [ . . . ] for some people, one thing’s the hook and then it leads to the other [ . . . ] (P1, Female, 75 years)

I actually started in the garden, [ . . . ] then I started walking [through] these doors and doing this course, that course [ . . . ] and it’s like “wow [ . . . ] I can do this!” (P5, Female, 44 years)

### Wider Community Benefits of The Grange and Suggestions for Improvement

In addition to individual benefits, many participants described an improved sense of community inclusion and physical appearance of the Grange Park housing estate resulting from the activities of The Grange (e.g., tree planting, litter picking and installation of artwork).

The people on the estate loved it, they were coming up to us when we were doing the litter picking. They were saying we were doing a great job, and how lovely the estate was looking now, and, to keep it up. People were actually coming out of their houses and saying how proud they were, that we were doing things like that. (P5, Female, 44 years)

Furthermore, most participants felt these activities contributed to a reduction in vandalism and crime on the estate.

A lot of people still think that we’re having that many problems, but [ . . . ], it’s not that bad anymore. I’ve been coming on my own. . . every single day now, and I haven’t been robbed, I haven’t been anything . . . (P6, Female, 26 years)

One of the participants, however, reported that vandalism was still a challenge on the estate.

I don’t like seeing the vandalism that there is sometimes . . . they [volunteers and staff] planted loads of lovely cherry trees not far from the building, and then they just all got broken off . . . they try to make the estate nicer and [people] vandalise things. (P2, Male, 52 years)

Although all participants reported that activities were open to everyone, others recognized some challenges to equity of provision, especially for those who were in employment.

There’s two broad groups of people on Grange Park Estate: people that don’t work and people that do work. So many things that happen here are for people that don’t work. If you do work, you don’t have access to the same things . . . (P1, Female, 75 years)

Participants also acknowledged that there were still many residents of the Estate who were not accessing the center. They suggested this was perhaps due to lack of awareness of the offer, or a lack of activities for certain groups (e.g., the elderly and older teenagers), rather than The Grange being seen as exclusive.

We need more [activities] for the teenagers, [ . . . ] I also think we need a bit more for the old people . . . (P5, Female, 44 years)

### Photovoice: Awareness Raising and Feelings of Empowerment

During the SSIs and FGD, many participants commented on how taking photographs facilitated much deeper thought about their perceptions, increasing their awareness about the activities and benefits of The Grange.

[taking and] seeing these pictures made me realise that there was more to the picture; like every day when you’re out, you don’t really notice, do you, but when you’re actually taking these pictures it just made you realise there was more to it . . .(P5, Female, 44 years [FGD])

At the photo-exhibition event, five participants (out of six) discussed their own photographs and recommendations resulting from the research with local “influential advocates” (further details in Table 2). Participants felt a strong sense of ownership over the research when they saw their ‘actual words’ in the captions accompanying their photos:

She’s actually used my words. (P5, Female, 44 years)

However, it is important to note that as part of the photovoice process, each participant reviewed and approved the commentaries prior to the photo-exhibition.

Last, the photo-exhibition seems to have positively affected participant empowerment through the facilitation of spontaneous discussions between participants and influential advocates. Participants felt proud to be able to influence the future of The Grange and in realizing that their input had been valued by those attending the event.

It’s good we’ve told you what needs to happen now, and people who matter have come to hear it [ . . . ] normally you do this research stuff, tell someone what you think, and nothing ever happens after it. (P2, Male, 52 years)

This is an important benefit that photovoice and the CBPR approach brought to the overall research process, by engaging participants to directly communicate their voices to the attention of influential stakeholders and raise their awareness about the issues identified, so that they can stimulate change (Hergenrather et al., 2009). Last, recognizing the value of the findings in the future development of The Grange, the staff made the photo-exhibition permanent within the hub so that the findings can be more widely disseminated in the community.

## Discussion

This study has demonstrated the value of using photovoice to reveal unique insights into the participants’ perceptions of community-led interventions and any perceived impact on health, well-being, and community inclusion. By adopting a CBPR approach, this study actively engaged a group of residents from a disadvantaged area, where high levels of health inequalities exist. Findings from the photo-exhibition and the experiences of being involved in the research demonstrate the ability of photovoice to affect participant empowerment through the facilitation of spontaneous conversation with influential advocates, and to drive positive change for the benefit of health, well-being, and community inclusion.

Alongside the positive physical and mental health benefits associated with gardening (Guitart et al., 2012), findings from this study suggest positive benefits on participation and community inclusion due to the interlink between the different activities of The Grange. This is consistent with previous studies where mixing of different generations and socioeconomic groups in community hubs and/or gardens resulted in improvements in social relations (Bagnall et al., 2018). Although our sample was homogeneous and did not allow us to explore this aspect in substantive depth, it did include different generations (one participant in her 20s and one in her 70s with others middle aged; Table 1). Combining multiple activities, can, however, create challenges of agenda matching between the community, providers, and funders (Pearson et al., 2010). In our study, the coproduction of the activities by users and volunteers, and the sense of equality between users and staff, created a sense of community inclusion that may have helped avoid tensions. In their recommendations, however, participants suggested to widen the reach of The Grange among Grange Park residents, such as more activities for teenagers and elderly people.

Community gardens have shown to cause tensions because of disputes over ownership and decision making (Kingsley et al., 2019; Webb, 2017), and some community members can feel excluded from community gardens. As we did not include nonusers of The Grange, it was not possible to explore this aspect among nonusers (Christensen et al., 2019; Spierings et al., 2018).

Community inclusion was defined as feeling part of “something bigger” than themselves. Through the Grange, participants developed a social network that supported them to overcome challenging times. However, cognizance should be given to some of the limitations of localized community projects as a Public Health Approach, which can support some (but not all) community members. It could be argued that The Grange offers a localized solution to address the consequences of wider social injustices with some evidence of success. However, this should not distract from the need to address the macro political factors that are causing inequality—such as food insecurity—which require urgent political attention (Marmot et al., 2020; Sosenko et al., 2019). Alongside the sense of social inclusion described by participants “within” The Grange, the activities of The Grange seem to have contributed to an improved sense of community spirit on the wider housing estate. In fact, improvements in the estate seemed to have led to a reduction in antisocial behavior and arson incidents (C. Jackson, personal communication, December 2019)—much in line with broken windows theory (Kelling & Wilson, 1984).

While this article does not explicitly discuss the pathways from involvement in The Grange to improved health and well-being and reducing health inequalities—which will be presented in a forthcoming article—the study findings seem to suggest that the sense of inclusion and friendships developed within The Grange may be more important factors for health improvement than the practical components of the intervention itself (Whitehead et al., 2016). The combination of activities and nuances of the ethos within which these are delivered make it, however, difficult to assess (Petticrew, 2011). It is difficult to distinguish if the perceived impact of The Grange may be due to an activity per se (e.g., gardening) or the combination of different factors (e.g., friendships), and how the context has influenced its implementation (Moore et al., 2019). In light of this, incorporating a systems thinking perspective into the evaluation of these interventions may help to explore the role of the context in shaping the intervention, and how changes in a part of the system (e.g., the culture) might affect other parts (e.g., activities delivered) and vice versa (Egan et al., 2019; Orton et al., 2017; Rutter et al., 2017).

### Strengths and Limitations

This study has highlighted the value of a community-led intervention in an area with high-socioeconomic deprivation, emphasizing the importance of equality among volunteers, staff, and users in decision making and running of the project, power balance, and not feeling judged as key determinants of engagement and success. Furthermore, the photo-exhibition seems to have positively affected participant empowerment through the facilitation of spontaneous discussions between participants and influential advocates. Through the photo exhibition, participants’ recommendations influenced local decision making by Blackpool Council and the management of The Grange, leading to further service developments. These include targeted activities for demographic groups not yet fully engaged in the project (including people aged 60+ years and 10–15 years); funding bids submitted by the Council and the project management to secure funding for the ongoing delivery of the project; and outreach to reach residents of the local housing estate that have not previously engaged with The Grange (C. Jackson, personal communication, February 2020).

Despite its strengths, the main limitations of this study relate to the homogeneous, small sample formed by frequent users of The Grange, which may have positively shaped the findings. Despite wanting a mix of users and nonusers, time restrictions of this study (conducted as part of a Master of Public Health dissertation), meant that the recruitment strategy—which used a gatekeeper that worked at the Grange—focused on recruiting users of The Grange. Recruiting residents who visited the center less frequently or not at all would have resulted in a more diverse study sample. Doing so would have enabled us to explore reasons for nonengagement including recommendations to widen The Grange audience from the nonusers’ perspectives. Also, financial constraints prevented the use of interpreters, which could have resulted in a more ethnically diverse sample. However, while the study participants may represent a somewhat homogeneous group, their demographics were representative of the local population of the Grange area (Public Health England, 2020).

### Implications for Theory, Policy, and Practice

Over the past years, there has been a call to understand the nuances of complexity of public health interventions (e.g., context and mechanisms of impact; Davis et al., 2019). Future interventions should incorporate some of the successful principles of The Grange including coproduction of the offer and ensuring a sense of equality among volunteers, staff, and users in decision making and running of the project. This can assist in making users and volunteers feel included, valued, and more confident. Moreover, these findings support the use of photovoice as a valuable method to explore community-led interventions that take place in a complex and changing context, supporting participants to feel heard and valued (Wang & Burris, 1997). However, incorporating photovoice in future mixed-methods evaluation that include economic evaluation may satisfy more fully the needs of both the local system and the wider research community.

In this study, participants emphasized the importance of “how’ services are delivered over the actual activities, which conflicts with often output driven U.K. Public Health commissioning (New Economics Foundation, 2014); commissioners might see greater health outcomes from service models, which prioritize true community empowerment. Photovoice offers an opportunity to influence health inequalities by using community voice to influence local decision making and engaging with underserved communities (Dahlgren & Whitehead, 1991). Local Government should consider the use of photovoice as a powerful tool to engage with community members to ensure that initiatives about health, well-being, and social inclusion are meaningful.

## Supplemental Material

sj-docx-1-heb-10.1177_10901981211009738 – Supplemental material for Residents’ Perceptions of a Community-Led Intervention on Health, Well-Being, and Community Inclusion Through Photovoice Click here for additional data file. Supplemental material, sj-docx-1-heb-10.1177_10901981211009738 for Residents’ Perceptions of a Community-Led Intervention on Health, Well-Being, and Community Inclusion Through Photovoice by Clare Jackson and Sara Ronzi in Health Education & Behavior

sj-docx-2-heb-10.1177_10901981211009738 – Supplemental material for Residents’ Perceptions of a Community-Led Intervention on Health, Well-Being, and Community Inclusion Through Photovoice Click here for additional data file. Supplemental material, sj-docx-2-heb-10.1177_10901981211009738 for Residents’ Perceptions of a Community-Led Intervention on Health, Well-Being, and Community Inclusion Through Photovoice by Clare Jackson and Sara Ronzi in Health Education & Behavior

sj-docx-3-heb-10.1177_10901981211009738 – Supplemental material for Residents’ Perceptions of a Community-Led Intervention on Health, Well-Being, and Community Inclusion Through Photovoice Click here for additional data file. Supplemental material, sj-docx-3-heb-10.1177_10901981211009738 for Residents’ Perceptions of a Community-Led Intervention on Health, Well-Being, and Community Inclusion Through Photovoice by Clare Jackson and Sara Ronzi in Health Education & Behavior

sj-docx-4-heb-10.1177_10901981211009738 – Supplemental material for Residents’ Perceptions of a Community-Led Intervention on Health, Well-Being, and Community Inclusion Through Photovoice Click here for additional data file. Supplemental material, sj-docx-4-heb-10.1177_10901981211009738 for Residents’ Perceptions of a Community-Led Intervention on Health, Well-Being, and Community Inclusion Through Photovoice by Clare Jackson and Sara Ronzi in Health Education & Behavior

sj-docx-5-heb-10.1177_10901981211009738 – Supplemental material for Residents’ Perceptions of a Community-Led Intervention on Health, Well-Being, and Community Inclusion Through Photovoice Click here for additional data file. Supplemental material, sj-docx-5-heb-10.1177_10901981211009738 for Residents’ Perceptions of a Community-Led Intervention on Health, Well-Being, and Community Inclusion Through Photovoice by Clare Jackson and Sara Ronzi in Health Education & Behavior

## References

[bibr1-10901981211009738] Abma T. Banks S. Cook T. Dias S. Madsen W. Springett J. Wright M. (2019). Participatory research for health and social well-being. Springer.

[bibr2-10901981211009738] Agee J. (2009). Developing qualitative research questions: A reflective process. International Journal of Qualitative Studies in Education, 22(4), 431–447. 10.1080/09518390902736512

[bibr3-10901981211009738] Bagnall A. South J. DiMartino S. Southby K. Pilkington G. Mitchell B. Pennington A. Corcoran R. (2018). Place, spaces, people and wellbeing: Full review. A systematic review of interventions to boost social relations through improvements in community infrastructure (places and spaces). What Works Wellbeing.

[bibr4-10901981211009738] Blackpool Council. (2017a). CLC business case. https://democracy.blackpool.gov.uk/documents/s20582/Appendix%202a%20CLC%20Business%20Case%20for%20jan%20Exec.pdf

[bibr5-10901981211009738] Blackpool Council. (2017b). Executive decision paper: City learning centre scheme. https://democracy.blackpool.gov.uk/documents/s20581/Item%202%20City%20Learning%20Centre%20Scheme.pdf

[bibr6-10901981211009738] Braun V. Clarke V . (2006). Using thematic analysis in psychology. Qualitative Research in Psychology, 3(2), 77–101. 10.1191/1478088706qp063oa

[bibr7-10901981211009738] Budig K. Diez J. Conde P. Sastre M. Hernan M. Franco M. (2018). Photovoice and empowerment: Evaluating the transformative potential of a participatory action research project. BMC Public Health, 18, Article 432. 10.1186/s12889-018-5335-7 PMC5879794 29609576

[bibr8-10901981211009738] Carlson E. D. Engebretson J. Chamberlain R. M. (2006). Photovoice as a social process of critical consciousness. Qualitative Health Research, 16(6), 836–852. 10.1177/1049732306287525 16760539

[bibr9-10901981211009738] Catalani C. Minkler M. (2010). Photovoice: A review of the literature in health and public health. Health Education & Behavior, 37(3), 424–451. 10.1177/1090198109342084 19797541

[bibr10-10901981211009738] Christensen S. Malberg Dyg P. Allenberg K. (2019). Urban community gardening, social capital, and” integration”: A mixed method exploration of urban” integration-gardening” in Copenhagen, Denmark. Local Environment, 24(3), 231–248. 10.1080/13549839.2018.1561655

[bibr11-10901981211009738] Craig P. Cooper C. Gunnell D. Haw S. Lawson K. Macintyre S. Ogilvie D. Petticrew M. Reeves B. Sutton M. Thompson S. (2012). Using natural experiments to evaluate population health interventions: New Medical Research Council guidance. Journal of Epidemiology and Community Health, 66(12), 1182–1186. 10.1136/jech-2011-200375 22577181 PMC3796763

[bibr12-10901981211009738] Craig P. Di Ruggiero E. Frohlich K. L. Mykhalovskiy E. White M. (2018). Taking account of context in population health intervention research: Guidance for producers, users and funders of research. NIHR Evaluation, Trials and Studies Coordinating Centre.

[bibr13-10901981211009738] Craig P. Katikireddi S. V. Leyland A. Popham P. (2017). Natural experiments: An overview of methods, approaches, and contributions to public health intervention research. Annual Review of Public Health, 38, 39–56. 10.1146/annurev-publhealth-031816-044327 PMC6485604 28125392

[bibr14-10901981211009738] Dahlgren G. Whitehead M. (1991). Policies and strategies to promote social equity in health. Institute for Futures Studies.

[bibr15-10901981211009738] Davis K. Minckas N. Bond V. Clark C. J. Colbourn T. Drabble S. J. Hesketh T. Hill Z. Morrison J. Mweemba O. Osrin D. Prost A. Seeley J. Shahmanesh M. Spindler E. J. Stern E. Turner K. M. Mannell J. (2019). Beyond interviews and focus groups: A framework for integrating innovative qualitative methods into randomised controlled trials of complex public health interventions. Trials, 20(1), Article 329. 10.1186/s13063-019-3439-8 PMC6555705 31171041

[bibr16-10901981211009738] Drew S. Guillemin M. (2014). From photographs to findings: Visual meaning-making and interpretive engagement in the analysis of participant-generated images. Visual Studies, 29(1), 54–67. 10.1080/1472586X.2014.862994

[bibr17-10901981211009738] Egan M. McGill E. Penney T. Anderson de Cuevas R. Er V. Orton L. White M. Lock K. Cummins S. Savona N. Whitehead M. Popay J. Smith R. Meier P. De Vocht F. Andreeva M. Rutter H. Petticrew M. (2019). NIHR SPHR guidance on systems approaches to local public health evaluation. Part 1: Introducing systems thinking. National Institute for Health Research School for Public Health Research.

[bibr18-10901981211009738] Evans-Agnew R. A. Rosemberg M.-A. S. (2016). Questioning photovoice research: Whose voice? Qualitative Health Research, 26(8), 1019–1030. 10.1177/1049732315624223 26786953

[bibr19-10901981211009738] Freire P. (1970). Pedagogy of the oppressed. Continuum.

[bibr20-10901981211009738] Freire P. (1974). Conscientisation. CrossCurrents, 24(1), 23–31.

[bibr21-10901981211009738] Groundwork. (2020). At the Grange. https://www.groundwork.org.uk/projects/at-the-grange/

[bibr22-10901981211009738] Guitart D. Pickering C. Byrne J. (2012). Past results and future directions in urban community gardens research. Urban Forestry & Urban Greening, 11(4), 364–373. 10.1016/j.ufug.2012.06.007

[bibr23-10901981211009738] Hacking J. M. Muller S. Buchan I. E. (2011). Trends in mortality from 1965 to 2008 across the English north-south divide: Comparative observational study. BMJ, 342. 10.1136/bmj.d508 PMC3039695 21325004

[bibr24-10901981211009738] Hawe P. Shiell A. Riley T. (2004). Complex interventions: How “out of control” can a randomised controlled trial be? BMJ, 328(7455), 1561–1563. 10.1136/bmj.328.7455.1561 15217878 PMC437159

[bibr25-10901981211009738] Hergenrather K. C. Rhodes S. D. Cowan C. A. Bardhoshi G. Pula S. (2009). Photovoice as community-based participatory research: A qualitative review. American Journal of Health Behavior, 33(6), 686–698. 10.5993/AJHB.33.6.6 19320617

[bibr26-10901981211009738] Hodgetts D. Chamberlain K. Radley A. (2007). Considering photographs never taken during photo-production projects. Qualitative Research in Psychology, 4(4), 263–280. 10.1080/14780880701583181

[bibr27-10901981211009738] Israel B. A. Coombe C. M. Cheezum R. R. Schulz A. J. McGranaghan R. J. Lichtenstein R. Reyes A. G. Clement J. Burris A. (2010). Community-based participatory research: A capacity-building approach for policy advocacy aimed at eliminating health disparities. American Journal of Public Health, 100(11), 2094–2102. 10.2105/AJPH.2009.170506 20864728 PMC2951933

[bibr28-10901981211009738] Kelling G. L. Wilson J. Q. (1984). Broken windows. The police and neighborhood safety. Atlantic. https://www.theatlantic.com/magazine/archive/1982/03/broken-windows/304465/

[bibr29-10901981211009738] Kingsley J. Foenander E. Bailey A. (2019). “You feel like you’re part of something bigger”: Exploring motivations for community garden participation in Melbourne, Australia. BMC Public Health, 19(1), 745. 10.1186/s12889-019-7108-3 31196077 PMC6567388

[bibr30-10901981211009738] Liebenberg L. (2018). Thinking critically about photovoice: Achieving empowerment and social change. International Journal of Qualitative Methods, 17, 1–9. 10.1177/1609406918757631

[bibr31-10901981211009738] Marmot M. Allen J. Boyce T. Goldblatt P. Morrison J. (2020). Health equity in England: The Marmot Review 10 years on. Institute of Health Equity. 10.1136/bmj.m693

[bibr32-10901981211009738] Minkler M. (2004). Ethical challenges for the “outside” researcher in community-based participatory research. Health Education & Behavior, 31(6), 684–697. 10.1177/1090198104269566 15539542

[bibr33-10901981211009738] Minkler M. (2005). Community-based research partnerships: Challenges and opportunities. Journal of Urban Health, 82(2), ii3–ii12. 10.1093/jurban/jti034 PMC3456439 15888635

[bibr34-10901981211009738] Moore G. F. Evans R. E. Hawkins J. Littlecott H. Melendez-Torres G. J. Bonell C. Murphy S. (2019). From complex social interventions to interventions in complex social systems: Future directions and unresolved questions for intervention development and evaluation. Evaluation, 25(1), 23–45. 10.1177/1356389018803219 30705608 PMC6330692

[bibr35-10901981211009738] Morgan A. Ziglio E. (2007). Revitalising the evidence base for public health: An assets model. International Journal of Health Promotion & Education, 14(2), 17–22. 10.1177/10253823070140020701x 17685075

[bibr36-10901981211009738] New Economics Foundation. (2014). Commissioning for outcomes and co-production. A New model for commissioning public services. https://neweconomics.org/2014/06/commissioning-outcomes-co-production

[bibr37-10901981211009738] NHS England. (2016). Engaging local people. A guide for local areas developing sustainability and transformation plans. https://www.england.nhs.uk/publication/engaging-local-people-a-guide-for-local-areas-developing-sustainability-and-transformation-plans/

[bibr38-10901981211009738] Nykiforuk C. I. Vallianatos H. Nieuwendyk L. M. (2011). Photovoice as a method for revealing community perceptions of the built and social environment. International Journal of Qualitative Methods, 10(2), 103–124. 10.1177/160940691101000201 27390573 PMC4933584

[bibr39-10901981211009738] O’Mara-Eves A. Brunton G. McDaid D. Oliver S. Kavanagh J. Jamal F. Matosevic T. Thomas J. (2013). Community engagement to reduce inequalities in health: A systematic review, meta-analysis and economic analysis. Public Health Research, 1(4). 10.3310/phr01040 25642563

[bibr40-10901981211009738] Orton L. Halliday E. Collins M. Egan M. Lewis S. Ponsford R. Powell K. Salway S. Townsend A. Whitehead M. Popay J. (2017). Putting context centre stage: Evidence from a systems evaluation of an area based empowerment initiative in England. Critical Public Health, 27(4), 477–489. 10.1080/09581596.2016.1250868

[bibr41-10901981211009738] Pearson S. Batty E. Cook B. Foden M. Knight-Fordham R. Peters J. (2010). Improving health outcomes in deprived communities: Evidence from the New Deal for Communities Programme. Department of Communities and Local Government.

[bibr42-10901981211009738] Petticrew M. (2011). When are complex interventions “complex?” When are simple interventions “simple?” European Journal of Public Health, 21(4), 397–398. 10.1093/eurpub/ckr084 21771736

[bibr43-10901981211009738] Public Health England. (2018). Guidance: Community-centred practice: Applying all our health. https://www.gov.uk/government/publications/community-centred-practice-applying-all-our-health

[bibr44-10901981211009738] Public Health England. (2020). Local authority health profile 2019. https://fingertips.phe.org.uk/static-reports/health-profiles/2019/e06000009.html?area-name=blackpool

[bibr45-10901981211009738] Public Health England, & NHS England. (2015). A guide to community-centred approaches for health and wellbeing. Full report. https://assets.publishing.service.gov.uk/government/uploads/system/uploads/attachment_data/file/768979/A_guide_to_community-centred_approaches_for_health_and_wellbeing__full_report_pdf

[bibr46-10901981211009738] Ronzi S. Pope D. Orton L. Bruce N. (2016). Using photovoice methods to explore older people’s perceptions of respect and social inclusion in cities: Opportunities, challenges and solutions. SSM: Population Health, 2, 732–744. 10.1016/j.ssmph.2016.09.004 29349184 PMC5757830

[bibr47-10901981211009738] Ronzi S. Puzzolo E. Hyseni L. Higgerson J. Stanistreet D. Hugo M. N. B. Bruce N. Pope D. (2019). Using photovoice methods as a community-based participatory research tool to advance uptake of clean cooking and improve health: The LPG adoption in Cameroon evaluation studies. Social Science & Medicine, 228, 30–40. 10.1016/j.socscimed.2019.02.044 PMC7081729 30875542

[bibr48-10901981211009738] Rutter H. Savona N. Glonti K. Bibby J. Cummins S. Finegood D. T. Greaves F. Harper L. Hawe P. Moore L. Petticrew M. Rehfuess E. Shiell A. Thomas J. White M. (2017). The need for a complex systems model of evidence for public health. Lancet, 390(10112), 2602–2604. 10.1016/S0140-6736(17)31267-9 28622953

[bibr49-10901981211009738] Spierings B. Van Liempt I. Maliepaard E. (2018). Ownership and membership: Practices and experiences of neighbourhood residents in the Wijsgeren Community Garden in Amsterdam. Tijdschrift voor Economische en Sociale Geografie, 109(5), 677–684. 10.1111/tesg.12337

[bibr50-10901981211009738] Sosenko F. Littlewood M. Bramley G. Fitzpatrick S. Blenkinsopp J. Wood J. (2019). State of hunger. A study of poverty and food insecurity in the UK. Trussell Trust.

[bibr51-10901981211009738] The King’s Fund. (2018). Local government spending on public health: Death by a thousand cuts. https://www.kingsfund.org.uk/blog/2018/01/local-government-spending-public-health-cuts

[bibr52-10901981211009738] Wallerstein N. (2006). What is the evidence on effectiveness of empowerment to improve health? WHO Regional Office for Europe.

[bibr53-10901981211009738] Wang C. Burris M. A. (1997). Photovoice: Concept, methodology, and use for participatory needs assessment. Health Education & Behavior, 24(3), 369–387. 10.1177/109019819702400309 9158980

[bibr54-10901981211009738] Wang C. C. Yi W. K. Tao Z. W. Carovano K. (1998). Photovoice as a participatory health promotion strategy. Health Promotion International, 13(1), 75–86. 10.1093/heapro/13.1.75

[bibr55-10901981211009738] Wang C. C. Redwood-Jones Y. A. (2001). Photovoice ethics: Perspectives from Flint Photovoice. Health Education & Behavior, 28(5), 560–572. 10.1177/109019810102800504 11575686

[bibr56-10901981211009738] Webb V. (2017). Good gardeners and bad plants: Governing health in community gardens. New Zealand Sociology, 32(2), 117–138.

[bibr57-10901981211009738] Whitehead M. (2014). Due north: Report of the inquiry on health equity for the north. University of Liverpool and Centre for Local Economic Strategies.

[bibr58-10901981211009738] Whitehead M. Pennington A. Orton L. Nayak S. Petticrew M. Sowden A. White M. (2016). How could differences in “control over destiny” lead to socio-economic inequalities in health? A synthesis of theories and pathways in the living environment. Health & Place, 39, 51–61. 10.1016/j.healthplace.2016.02.002 26986982

