# Supplementary material for: Residents’ Perceptions of a Community-Led Intervention on Health, Well-Being, and Community Inclusion Through Photovoice
Source: Health Educ Behav. 2021 May 21;48(6):783–94. doi: 10.1177/10901981211009738 (PMC8581723; doi:10.1177/10901981211009738)
Supplement: sj-docx-1-heb-10.1177_10901981211009738 – Supplemental material for Residents’ Perceptions of a Community-Led Intervention on Health, Well-Being, and Community Inclusion Through Photovoice [file sj-docx-1-heb-10.1177_10901981211009738.docx]

**Appendix A**

Overview of main activities at The Grange

| Community Library  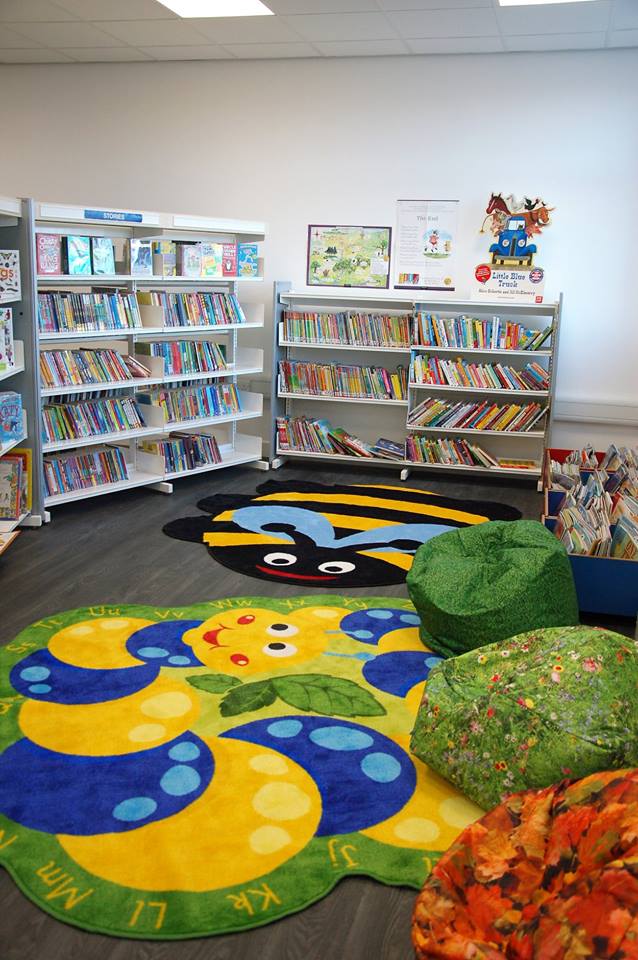 | Community Farm  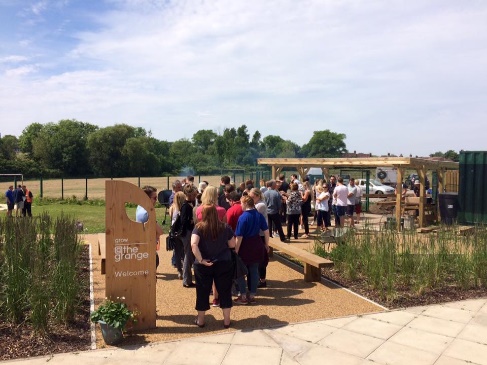 |
| --- | --- |
| Grow and Eat Activities  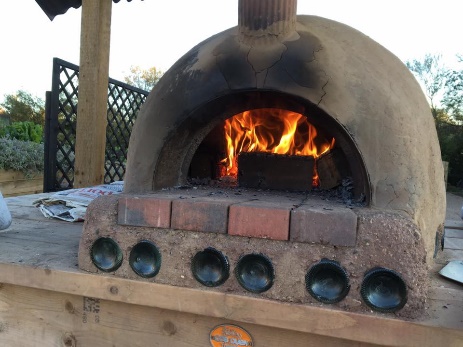 | **Community Shop**  **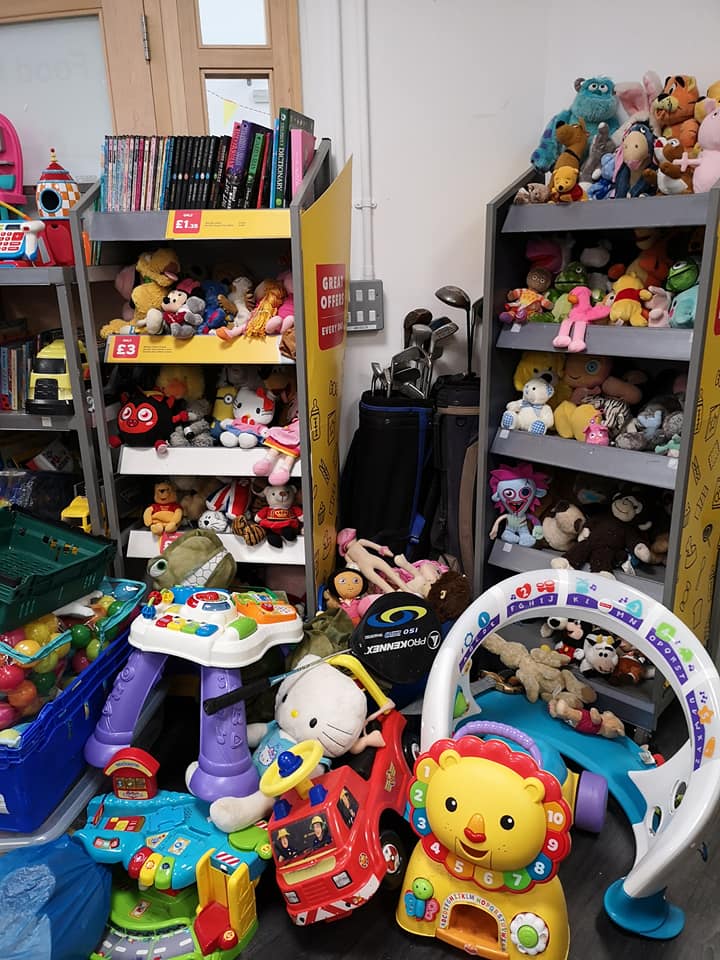** |
| Community Workshops  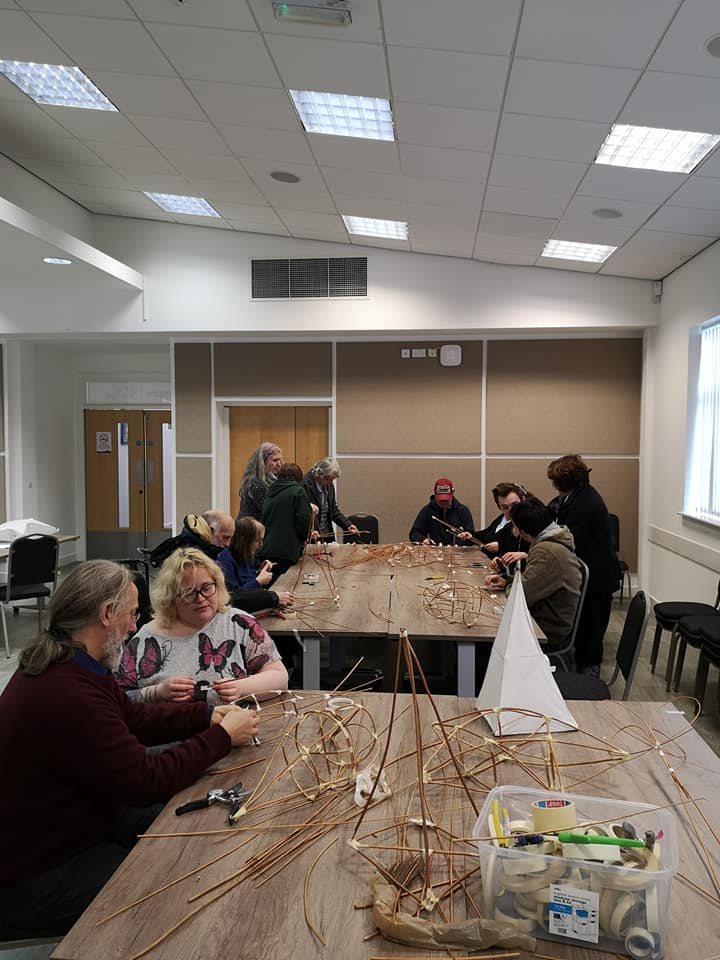 | **Community Spaces**  **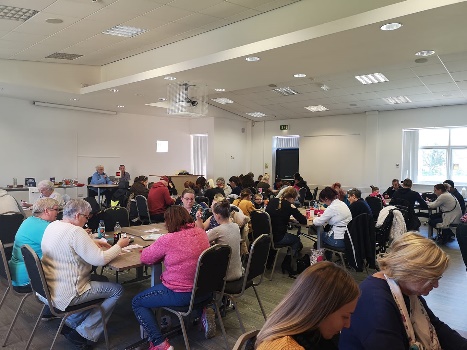** |
| Community gardening  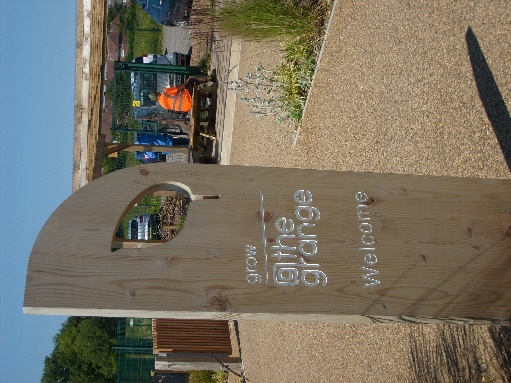 |  |
