# Supplementary material for: Residents’ Perceptions of a Community-Led Intervention on Health, Well-Being, and Community Inclusion Through Photovoice
Source: Health Educ Behav. 2021 May 21;48(6):783–94. doi: 10.1177/10901981211009738 (PMC8581723; doi:10.1177/10901981211009738)
Supplement: sj-docx-2-heb-10.1177_10901981211009738 – Supplemental material for Residents’ Perceptions of a Community-Led Intervention on Health, Well-Being, and Community Inclusion Through Photovoice [file sj-docx-2-heb-10.1177_10901981211009738.docx]

**Appendix B**

**Individual Semi-Structured Interview - Topic Guide**

Section 1: Exploring the meaning of the photographs. Questions to be repeated for each photograph.

1. Tell me why you took this photograph.

- *Why do you want to share this photograph?*
- *Why is it important for you?*

The SHOWeD technique (Wang et al. 1998, Wang & Burris 1997)

- - *What do you See here?*
  - *What's really Happening here?*
  - *How does this relate to Our lives?*
  - *Why does this problem, concern, or strength Exist?*
  - *What can we Do about it?*

1. In what way does the image in this photograph affect/relate to your health and wellbeing?
   - *What does health and wellbeing mean to you?*

- *Mental health*
- *Physical health*
- *General wellbeing*
  - *Wider determinants of health (housing, amenities, transport, education etc)*

1. In what way does the image in this photograph affect/relate to the way you feel part of the community on Grange Park?
   - *People*
   - *Places*
   - *Services*
   - *Infrastructure/wider determinants (housing, amenities, transport etc)*
   - *How have things changed/stayed the same since the opening of The Grange?*

 Section 2: Exploring wider perceptions of The Grange and its impact on health, wellbeing, and community inclusion.

1. Can you give an example of a time when you felt connected/part of the Grange Park Community?
   - *Social inclusion*
   - *People*
   - *Places*
   - *Services*
   - *Infrastructure/wider determinants (housing, amenities, transport etc)*
   - *What impact has The Grange had on…?*
   - *Does everyone who lives in Grange Park have the same/different experiences?*

1. Would you like to give me any example of a time when you found difficult/struggled feeling part of the Grange Park community?
   - *People*
   - *Places*
   - *Services*
   - *Infrastructure/wider determinants (housing, amenities, transport etc)*
   - *What impact has The Grange had?*
   - *Does everyone who lives in Grange Park have the same/different experiences?*

1. Can you give me any examples of things about Grange Park that you think help you to be healthy and feel good?
   - *People*
   - *Places*
   - *Services*
   - *Infrastructure/wider determinants (housing, amenities, transport etc)*
   - *What impact has The Grange had?*
2. Can you give me any examples of things about Grange Park that you think make it difficult to be healthy and feel good?
   - *People*
   - *Places*
   - *Services*
   - *Infrastructure/wider determinants (housing, amenities, transport etc)*
   - *What impact has The Grange had?*

Section 3: Summarising and Concluding

1. Is there anything that you would do/should be done to improve health and wellbeing on Grange Park?

- *Is there anything you would change about The Grange? If so, what?*

1. Is there anything that you would do/should be done to improve community inclusion on Grange Park?
   - *Is there anything you would change about The Grange? If so, what?*

1. Are there any photographs that you might have wanted to take but you did not?
   - *If yes, can you tell me more about the aspect(s) you wanted to photograph?*
   - *What prevented you from taking that photograph? (E.g. lack of time…)*
   - *Is there any challenge you experience in taking photographs?*
   - *How are you finding the project so far?*
   - *How did you find the photographic task?*
   - *What did you like most and least about taking photographs and being involved in this project?*
