# Supplementary material for: Residents’ Perceptions of a Community-Led Intervention on Health, Well-Being, and Community Inclusion Through Photovoice
Source: Health Educ Behav. 2021 May 21;48(6):783–94. doi: 10.1177/10901981211009738 (PMC8581723; doi:10.1177/10901981211009738)
Supplement: sj-docx-3-heb-10.1177_10901981211009738 – Supplemental material for Residents’ Perceptions of a Community-Led Intervention on Health, Well-Being, and Community Inclusion Through Photovoice [file sj-docx-3-heb-10.1177_10901981211009738.docx]

## **Appendix C**

Analytic framework developed in this study.

| Sub-themes | Codes |
| --- | --- |
| Feelings | Belonging  Comfortable  Pride  Feeling at ease  Achievement  Confidence  Ownership  Respect  Responsibility  Non-judgemental  Acceptance  Recognition  Escapism |
| Community | Company  Intergenerational  Pleasure in community inclusion  Places and spaces for community  Real vs. virtual community  Community spirit  Reciprocity  Social connection  Community benefit  Unifying  Families  Friendship |
| The Grange | Inside/outside link  Welcoming  First visit  Local/distance  Ease of access  Staff  Community navigators  Information hub  Atmosphere and culture |
| Dietary | Food purchase  Seasonal eating  Diet/healthy eating  Food deserts |
| Problems and Challenges | Vandalism  Drugs/alcohol/substance misuse  Poverty  Transience  Transport  Complexity of services and systems |
| Activities | Get out of the house  Meaningful activity  Gardening for emotional wellbeing  Exercise  Learning  Volunteering  Pizza oven |
| Concepts | Advocacy  Identity  Equality  Inclusion  Social determinants of health  Power  Social value  Trust |
