# Supplementary material for: Residents’ Perceptions of a Community-Led Intervention on Health, Well-Being, and Community Inclusion Through Photovoice
Source: Health Educ Behav. 2021 May 21;48(6):783–94. doi: 10.1177/10901981211009738 (PMC8581723; doi:10.1177/10901981211009738)
Supplement: sj-docx-4-heb-10.1177_10901981211009738 – Supplemental material for Residents’ Perceptions of a Community-Led Intervention on Health, Well-Being, and Community Inclusion Through Photovoice [file sj-docx-4-heb-10.1177_10901981211009738.docx]

# **Appendix D**

Selected examples of connecting participant-generated themes and the sub-themes/themes that emerged from the thematic analysis.

| Participant Generated Theme(s) in the focus group discussion (Phase 4) | Photograph | Photograph title developed by participant | Accompanying Quotation | Theme emerging from the Thematic Analysis |
| --- | --- | --- | --- | --- |
| Forget your troubles and feel safe, Gardening as a therapy | 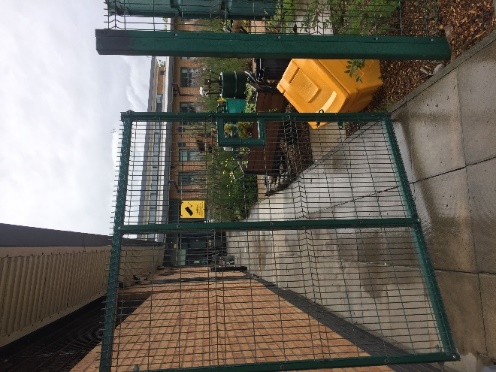 | The Back Gate | *“I suffer from depression, and it’s a big thing for me, volunteering The Grange. And as soon as I walk through that back gate, I feel perfectly safe and happy and all my troubles seem to have been lifted from me...”* (P3, SSI, F, 59yrs) | Escapism, safe spaces and identity |
| Gardening as a therapy; Environmental sustainability; Involving the community in meaningful activities | 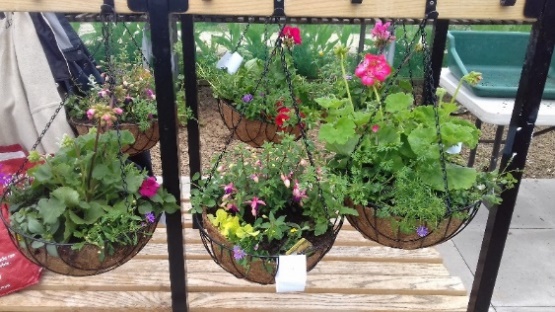 | The Grange hanging basket workshop for Grange Park residents | *"When you walk around, and you can see them...it does look lovely!" (*P5, SSI, F, 44yrs) | Wider community benefits of The Grange and suggestions to widen the offer |
| Focal points and gathering places; Not being judged | 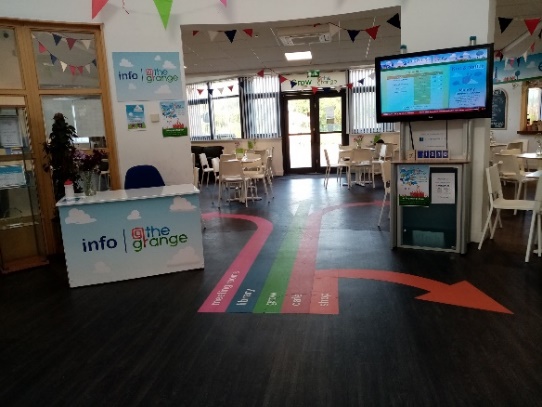 | Volunteers play a pivotal role in helping the community to navigate the offer The Grange | *“If we see someone who is in need of help or assistance […] then it’s our job to make sure that they know where they're going”*  *(*P6, SSI, F, 26yrs) | Giving and receiving help and support through ‘bottom up’ community development |
| Focal points and gathering places; ‘Forget your troubles and feel safe’; Involving the community in meaningful activities | 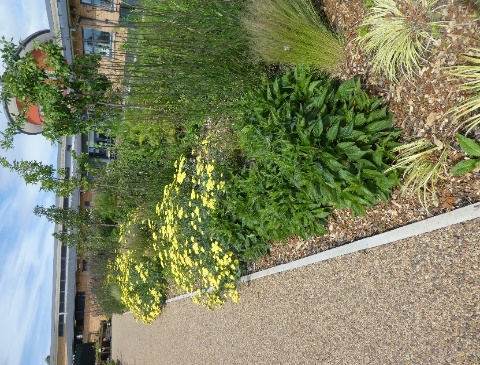 | The enticing gardens The Grange | *“The inside encourages you to go outside…you could see it through the windows, and it looks interesting… so, for some people, one thing’s the hook and then it leads to the other…” (*P1, SSI, F, 75yrs) | The relationship between hub and garden and its perceived impact on health, wellbeing and community inclusion |
