# Supplementary material for: Residents’ Perceptions of a Community-Led Intervention on Health, Well-Being, and Community Inclusion Through Photovoice
Source: Health Educ Behav. 2021 May 21;48(6):783–94. doi: 10.1177/10901981211009738 (PMC8581723; doi:10.1177/10901981211009738)
Supplement: sj-docx-5-heb-10.1177_10901981211009738 – Supplemental material for Residents’ Perceptions of a Community-Led Intervention on Health, Well-Being, and Community Inclusion Through Photovoice [file sj-docx-5-heb-10.1177_10901981211009738.docx]

## **Appendix E**

Participant-generated themes and number of photographs classified under each theme

| **Theme** | **Photographs assigned by participants** |
| --- | --- |
| Focal points and gathering places | 6 |
| Gardening as a therapy | 6 |
| ‘Forget your troubles and feel safe’ | 7 |
| Family and intergenerational activities | 3 |
| Not being judged | 4 |
| Environmental sustainability | 5 |
| Involving the community in meaningful activities | 5 |

*Some photographs feature in more than one theme
